# Translation of *ABCE1* Is Tightly Regulated by Upstream Open Reading Frames in Human Colorectal Cells

**DOI:** 10.3390/biomedicines9080911

**Published:** 2021-07-29

**Authors:** Joana Silva, Pedro Nina, Luísa Romão

**Affiliations:** 1 Departamento de Genética Humana, Instituto Nacional de Saúde Doutor Ricardo Jorge, 1649-016 Lisboa, Portugal; joana.filipap.silva@gmail.com (J.S.); pedro.lopes@insa.min-saude.pt (P.N.); 2 Instituto de Biossistemas e Ciências Integrativas (BioISI), Faculdade de Ciências, Universidade de Lisboa, 1749-016 Lisboa, Portugal

**Keywords:** translational regulation, uORFs, ABCE1, colorectal cancer

## Abstract

ATP-binding cassette subfamily E member 1 (ABCE1) belongs to the ABC protein family of transporters; however, it does not behave as a drug transporter. Instead, ABCE1 actively participates in different stages of translation and is also associated with oncogenic functions. Ribosome profiling analysis in colorectal cancer cells has revealed a high ribosome occupancy in the human *ABCE1* mRNA 5′-leader sequence, indicating the presence of translatable upstream open reading frames (uORFs). These *cis*-acting translational regulatory elements usually act as repressors of translation of the main coding sequence. In the present study, we dissect the regulatory function of the five AUG and five non-AUG uORFs identified in the human *ABCE1* mRNA 5′-leader sequence. We show that the expression of the main coding sequence is tightly regulated by the *ABCE1* AUG uORFs in colorectal cells. Our results are consistent with a model wherein uORF1 is efficiently translated, behaving as a barrier to downstream uORF translation. The few ribosomes that can bypass uORF1 (and/or uORF2) must probably initiate at the inhibitory uORF3 or uORF5 that efficiently repress translation of the main ORF. This inhibitory property is slightly overcome in conditions of endoplasmic reticulum stress. In addition, we observed that these potent translation-inhibitory AUG uORFs function equally in cancer and in non-tumorigenic colorectal cells, which is consistent with a lack of oncogenic function. In conclusion, we establish human *ABCE1* as an additional example of uORF-mediated translational regulation and that this tight regulation contributes to control ABCE1 protein levels in different cell environments.

## 1. Introduction

Gene expression is extremely regulated at the level of mRNA translation, especially at its initiation step, where the initiation codon is identified and decoded [1,2]. Translational regulation involves a large number of protein factors and regulatory mRNA sequence elements predominantly located in their 5′ and 3′-leader sequences (also known as untranslated regions (UTRs)) [3]. These include small structural elements, internal ribosome entry sites, and regulatory upstream open reading frames (uORFs) [4,5]. With the wide application of ribosome profiling (Ribo-Seq), it was possible to detect a significant ribosomal occupancy in mRNA regions that were thought to be non-coding, including the 5′-leader sequences (i.e., 5′UTRs), which is consistent with the widespread presence of translatable uORFs among the transcriptome [6,7,8]. Bioinformatics studies estimate that ~50% to ~60% of human transcripts have at least one uORF [9,10]. Indeed, uORFs are frequently present in transcription factors, cellular receptors, oncogenes, and transcripts with a role in stress response, cell growth, and differentiation control [11,12,13,14]. uORFs are typically described as repressors of translation initiation at the main coding sequence under normal cellular conditions [4,9,13]. Translational repression can be achieved by ribosome dissociation and consequent ribosome recycling after uORF translation or by ribosome stalling of the elongating/terminating ribosomes during uORF translation [1,4]. In addition, ribosomal stalling at the uORF termination codon can trigger nonsense-mediated mRNA decay (NMD) since the uORF stop codon can be recognized as a premature termination codon [15,16,17]. Furthermore, translation of uORFs may also titrate translation initiation factors [1]. The uORF-encoded peptides can also regulate main ORF expression acting as *cis*- or *trans*-repressors [18,19,20,21]. In the end, the repressive function of uORFs on the main coding sequence translation depends on several factors, such as the number and length of the uORFs, the distance between a uORF and the downstream coding sequence, and the uORF start codon and its context [9,10,13,22,23,24,25]. Other *cis*-acting elements can also affect uORF translation efficiency. These include the secondary structures in the 5′-leader sequence, which can affect ribosomal recognition of the uORF initiation codon [25,26]. More recently, it has also been shown that the presence of the RNA modification *N*^6^-methyladenosine (m^6^A), or the presence of exon-exon junctions and consequently exon junction complexes, in the mRNA 5′-leader sequence can promote uORF translation [27,28]. Recent data have revealed that non-AUGs can also function as initiation codons of uORFs, their usage also being affected by the initiation codon sequence context and mRNA secondary structures [26,29,30]. Moreover, among the uORFs identified by Ribo-Seq, the ones initiating in non-canonical start codons are more frequent compared to the AUG-initiating uORFs [7,31].

Under cellular stress, such as the endoplasmic reticulum (ER) stress, global protein synthesis is inhibited in part by decreasing the amount of available ternary complex through phosphorylation of the alpha-subunit of the initiation factor eIF2 [32,33,34]. Nevertheless, these conditions promote the selective synthesis of specific proteins, many of which are associated with stress adaptive processes [1,35,36]. Increased synthesis of these proteins depends on the presence of uORFs in the corresponding mRNAs [1,37]. When the ternary complex is scarce due to cellular stress, the scanning ribosome is less likely to acquire an active ternary complex before reaching the inhibitory uORF, and scanning continues downstream toward the main initiation codon, allowing higher translational efficiency of the main ORF [1,35,36]. However, not all the uORF-containing transcripts are translationally induced by cellular stress, indicating that uORF-mediated translational regulation is transcript-specific [37]. Nonetheless, the importance of uORF-mediated translational regulation is well illustrated by the fact that mutations that introduce or disrupt a uORF can cause human disease, including cancer [24,38,39]. Thus, identifying when and how uORFs function is essential to understand the etiology of many human diseases.

The human ATP-binding cassette (ABC) protein family is the largest family of transporters, being ABCE1 protein the only member of the subfamily E (ABCE) [40,41]. *ABCE1* gene is localized in chromosome 4, at position 4q31, and its main ORF encodes a 599 amino-acid protein (~67 kDa), found in the nucleus and in the cytoplasm of mammalian cells [42,43,44]. ABCE1 protein was first described as an inhibitor of the 2-5A/RNase L system, a central pathway of interferon action [43,45,46]. Additionally, it was demonstrated that ABCE1 has relevant functions transversally in mRNA translation, being shown a crucial role of ABCE1 in translation initiation, translation termination, and recycling [42,47,48,49,50]. The chromosomic localization of the *ABCE1* gene is a fragile site already associated with structural rearrangements in cancers [51]. In fact, ABCE1 overexpression has been associated with the tumorigenic process of several human cancers, for instance, lung cancer [52,53,54], breast cancer [55], esophageal cancer [56], ovarian cancer [57], and glioma [58]. On the other hand, *ABCE1* mRNA is ubiquitously expressed both in normal and in cancer colorectal cells [59]. In a contradicting study by Hlavata and co-workers, an upregulation of *ABCE1* mRNA in a pool of colorectal cancers was shown [60].

Several Ribo-Seq studies performed in different cell lines have revealed a wide ribosome occupancy in the 5′-leader sequence of the human *ABCE1* transcript [36,61,62]. Inclusive, in the colorectal carcinoma cell line HCT116, it was identified several uORFs starting with non-canonical initiation codons [61]. Interestingly, in a bioinformatic study, it was predicted the existence of two AUG uORFs within the human *ABCE1* 5′-leader sequence [63]. These data suggest that the *ABCE1* transcript may itself be subjected to a high translational regulation mediated by uORFs. Taking into account the important functions of the ABCE1 protein and the unknown role of its uORFs, our aim was to study the function of *ABCE1* uORFs in its translational control in colorectal cancer cells. Here, we show that the human *ABCE1* 5′-leader sequence contains five functional AUG uORFs, from which uORF3 (or uORF4 as they are in frame and overlapped) and uORF5 function as repressors of the main ORF translation, while uORF1, and to a less extent uORF2, function as regulators of the downstream repressive uORFs. On the contrary, the five non-AUG uORFs identified do not exhibit significant repression activity. We also show that this uORF-mediated translational regulation of *ABCE1* responds to ER stress conditions. However, it works equally in cancer and in non-tumorigenic colorectal cells, which is consistent with a lack of oncogenic function. In conclusion, we establish human *ABCE1* as an additional example of uORF-mediated translational regulation.

## 2. Experimental Section

### 2.1. Reporter Vectors

Synthesis by overlap extension (SOEing) polymerase chain reaction (PCR) was used to obtain a firefly luciferase (FLuc) reporter plasmid carrying the 515 nt-long 5′-leader sequence of the human *ABCE1* transcript (5′-leader sequence information based in the Ensembl transcript ENST00000296577, assembly GRCh37). In the first PCR, the *ABCE1* 5′ untranslated sequence was amplified from a cDNA sample obtained from NCM460 cells (non-tumorigenic colorectal cell line) with the primers #1 (with a linker for the HindIII restriction site; Table S1) and #2 (with a linker for the 5′-end of the FLuc ORF; Table S1). In the second PCR, the 5′-part of the FLuc ORF (region comprised between HindIII restriction site and a few nucleotides downstream of the BsrGI restriction site) was amplified from the pGL2-enhancer vector (Promega; Madison, WI, USA) already modified to contain the human cytomegalovirus (hCMV) promotor (called pGL2-FLuc or empty vector [64]), using the primers #3 (with a linker for the 3′-end of the *ABCE1* 5′-leader sequence; Table S1) and #4 (a primer designed for the FLuc ORF downstream of the BsrGI restriction site; Table S1). With a third PCR, SOEing PCR, and using the primers #1 and #4, the *ABCE1* 5′-leader sequence fragment was fused upstream of the FLuc ORF in a way that the FLuc AUG replaces the AUG of the transcript main ORF.

The SOEing PCR product and the pGL2-FLuc plasmid were digested with HindIII (Fermentas; Waltham, MA, USA) and BsrGI (New England Biolabs; Ipswich, MA, USA), and the two products were ligated with the T4 DNA ligase (NZYTech; Lisboa, Portugal) as instructed by the manufacturer, forming the ABCE1_5′UTR construct (5′UTR as simplification of 5′-leader sequence). NZY5α competent cells (NZYTech) were transformed with the ABCE1_5′UTR construct to generate clones of this vector. NZYMiniprep kit (NZYTech) was used for plasmid DNA (pDNA) extraction, following the manufacturer’s instructions and the sequence of the reporter plasmid confirmed by sequencing.

All the following constructs used in this study were obtained by site-directed mutagenesis following a standard protocol. Briefly, 30 ng of template (pDNA) were amplified in a PCR reaction using lengthened primers containing the genetic alteration of interest and a proof-reading enzyme (NZYSpeedy Proof DNA polymerase; NZYTech). The resulting PCR product was digested with DpnI (Thermo Fisher Scientific; Waltham, MA, USA), NZY5α competent cells transformed, and pDNA extracted. All the mutagenesis constructs were again confirmed by sequencing.

Thus, by site-directed mutagenesis and using the ABCE1_5′UTR plasmid, each uAUG was altered from an ATG to TTG (primers #5 to #14 in Table S1), obtaining constructs with: (i) only one functional AUG uORF (called: uORF1, uORF2, uORF3, uORF4, and uORF5); (ii) only one mutated AUG uORF (called: “no uORF1”, “no uORF2”, “no uORF3”, “no uORF4”, and “no uORF5”); (iii) different combinations of functional/mutated uORFs where uORF1 and uORF2 are always deleted (called: “no uORF1+2”, “no uORF1+2+3”, “no uORF1+2+4”, and “no uORF1+2+5”); and (iv) none of the AUG uORFs functional (called: “no AUG uORFs”). The construct “no AUG uORFs” was also subjected to successive mutagenesis to alter the non-canonical initiation codons from CTG/GTG to GCG (primers #1 to #16 in Table S2), obtaining constructs with only one functional non-AUG uORF (called uORF6, uORF7, uORF8, uORF9, and uORF10) or without functional non-AUG uORFs (called “no uORFs”). Additionally, the CTG/GTG initiation codons of each one of the non-canonical uORFs were altered to an ATG by site-directed mutagenesis using the “no AUG uORFs” construct (primers #17 to #26 in Table S2), resulting in the constructs uORF6_AUG, uORF7_AUG, uORF8_AUG, uORF9_AUG, and uORF10_AUG.

Reporter plasmids were also obtained in which the uORF sequence is fused in frame with the FLuc ORF. For that, the following mutagenesis reactions were performed on the constructs with only one functional AUG uORF: (i) for uORF3 and uORF4 constructs, 1 nucleotide (T) was deleted in the intercistronic space between the shared uORF’s stop codon and the beginning of the FLuc ORF (primers #15 and #16 in Table S1); and (ii) for the five uORF-individual constructs, each in-frame stop codon was mutated from TGA to GGA (primers #17 to #26 in Table S1). The resulting constructs were called: Fused_uORF1_AUG, Fused_uORF2_AUG, Fused_uORF3_AUG, Fused_uORF4_AUG, and Fused_uORF5_AUG. Additionally, the Kozak consensus sequence of each uAUG in the constructs uORF1-5 was mutated to an optimal Kozak consensus with the sequence ACCATGG (primers #27 to #36 in Table S1). Those constructs were called: Optimal_uAUG1, Optimal_uAUG2, Optimal_uAUG3, Optimal_uAUG4, and Optimal_uAUG5. In addition, to obtain out-of-frame and overlapped AUG uORFs relative to the FLuc ORF, series of sequential mutagenesis were made: (i) in uORF1, uORF2 and uORF5 constructs, only 1 nucleotide (T) was deleted in the intercistronic space between the uORFs’ sequences and the beginning of the FLuc ORF (primers #37 and #38 in Table S1); and, (ii) for all the five uORF-individual constructs, each in-frame stop codon was mutated from TGA to GGA (primers #17 to #26 in Table 1). Those constructs were named: “mut stops_uORF1”, “mut stops_uORF2”, “mut stop_uORF3”, “mut stop_uORF4”, and “mut stops_uORF5”.

### 2.2. Cell Culture

The human colorectal carcinoma cell line HCT116, two human colorectal adenocarcinoma cell lines DLD-1 and SW480, were grown in Dulbecco’s Modified Eagle Medium (DMEM; Gibco, Thermo Fisher Scientific) supplemented with 10% (*v/v*) fetal bovine serum (FBS; Gibco, Thermo Fisher Scientific). The non-tumorigenic colorectal cell line NCM460 and two colorectal adenocarcinoma cell lines CaCo-2 and HT-29 were grown in Roswell Park Memorial Institute 1640 (RPMI; Gibco, Thermo Fisher Scientific, Waltham, MA, USA) supplemented with 10% (*v/v*) FBS. Cells were maintained in an incubator at 37 °C, in a humidifier atmosphere with 5% (*v/v*) CO_2_.

### 2.3. Cell Transfections and Treatments

HCT116 and NCM460 cells were seeded in 35 mm plates at 70–80% confluence and 24 h after, were transiently co-transfected with 1.5 µg of each constructed plasmid, and 0.5 µg of pRL-TK vector (Promega), which expresses *Renilla* luciferase (RLuc), using Lipofectamine 2000 Transfection Reagent (Invitrogen; Waltham, MA, USA) and following the manufacturer’s instructions. Briefly, in a first solution, the amount of each reporter plasmid was diluted in 250 µL of Opti-MEM medium (Gibco, Thermo Fisher Scientific, Waltham, MA, USA), and in a second solution, 4 µL of Lipofectamine 2000 were also diluted in 250 µL of Opti-MEM. Both solutions were mixed and rest for at least 20 min at room temperature. In the meantime, the culture medium was changed to fresh growth media, where afterward, the mixture above was added dropwise, and cells were incubated for 24 h at 37 °C, in a humidifier atmosphere with 5% (*v/v*) CO_2_. In stress studies, HCT116 cells were also transiently co-transfected, and 4 h later, treated with 4 µM of thapsigargin (Tg; Enzo Life Sciences; Farmingdale, NY, USA) or dimethyl sulfoxide (DMSO; Sigma; St. Louis, MO, USA), the vehicle control of thapsigargin, for 24 h at 37 °C, in a humidifier atmosphere with 5% (*v/v*) CO_2_. In either cases, cells were harvested for further analyses.

For transfections with siRNAs, HCT116, and NCM460 cells were seeded at 30–40% confluence in 96-well plates and after 24 h, 40 pmol of the siRNA for *ABCE1* (5′-UCAUCAAACCUCAAUAUGU-3′), or the siRNA for Luciferase (*LUC*; 5′-CGUACGCGGAAUACUUCGA-3′), used as a control condition (Thermo Fisher Scientific), were transiently transfected using Lipofectamine 2000, as above. Twenty-four hours after transfection, the knockdown was reinforced with 40 pmol of each corresponding siRNA. Cells were harvested 48 h after the first siRNA transfection, and cleared cell lysates were used for Western blot analysis. For cell viability assays, cells were kept adherent to the plate.

### 2.4. Luminometry Assays

Cells were washed with 1 mL of pre-chilled 1× (*v/v*) phosphate-buffered saline (PBS 1×), lysed in 100 µL of 1× (*v/v*) passive lysis buffer (PLB 1×; Promega) and centrifuged at maximum speed to obtain cleared cell lysates. FLuc and RLuc activity was assessed using the Dual-Luciferase^®^ Reporter Assay System (Promega), according to the manufacturer’s instructions, on a GloMax^®^ 96 Microplate Luminometer (Promega). Briefly, 10 µL of the cleared cell lysates were plated in a white 96-well plate. First, we measured the luminescence signal of the FLuc reporter by adding 40 µL of the Luciferase Assay Reagent (containing the firefly luciferase substrate) to each sample. Then, RLuc reporter luminescence was sequentially quantified from the same sample by adding 40 µL of the Stop & Glo^®^ Reagent that first quenches the FLuc luminescence reaction and contains the substrate for the RLuc reaction. The collected data were expressed in arbitrary light units. Luciferase activity of each condition is obtained by normalizing FLuc to RLuc luminescence.

### 2.5. Western Blotting

From the cleared cell lysates obtained after cells were washed with 1 mL of pre-chilled PBS 1×, lysed in 100 µL of PLB 1× and centrifuged at maximum speed, we used the correspondent volume to 30 µg of total protein for Western blot analysis, previously quantified with NZYBradford reagent (NZYTech), as instructed by the manufacturer, in the NanoDrop 1000 (Thermo Fisher Scientific, Waltham, MA, USA). Denaturation of samples was performed by adding 5× (*v/v*) sodium dodecyl sulfate (SDS) sample buffer (NZYTech) to the protein sample and heating at 95 °C for 15 min. Afterward, samples were resolved by SDS-polyacrylamide gel electrophoresis (SDS-PAGE) in a 10 or 12% acrylamide/bisacrylamide gel and then transferred to methanol pre-activated polyvinyldene difluoride (PVDF) membranes (Bio-Rad; Hercules, CA, USA). Depending on the primary antibody, membranes were blocked in 5% (*w/v*) non-fat dry milk or bovine serum albumin (BSA; Sigma, St. Louis, MO, USA), diluted in 1× (*v/v*) Tris-buffered saline (TBS 1×) supplemented with 0.05% (*v/v*) Triton x-100 (Sigma) or Tween 20 (Sigma). Membranes were blotted overnight (o/n), at 4 °C, with shacking, with the following primary antibody solutions: mouse anti-α-tubulin (Sigma) diluted 1:50,000 in 5% (*w/v*) non-fat milk in 0.05% (*v/v*) Triton-TBS 1×; rabbit anti-Pospho-eIF2α (Thermo Fisher Scientific, Waltham, MA, USA) diluted 1:250 in 5% (*w/v*) BSA in 0.05% (*v/v*) Tween-TBS 1×; goat anti-Firefly Luciferase (Abcam; Cambridge, UK) diluted 1:250 in the solution 1 for primary antibodies from the SignalBoost™ Immunoreaction Enhancer Kit (Merck Milipore; Kenilworth, NJ, USA); and rabbit anti-ABCE1 (Abcam) diluted 1:1000 in 5% (*w/v*) non-fat milk in 0.05% (*v/v*) Tween-TBS 1×. Detection was performed at room temperature, with shaking, using secondary peroxidase-conjugated anti-mouse IgG (Bio-Rad), anti-rabbit IgG (Bio-Rad) or anti-goat IgG (Bio-Rad), diluted 1:4000, 1:3000, or 1:5000, respectively, in 5% (*w/v*) non-fat milk in the corresponding buffers, followed by enhanced chemiluminescence.

As in the case of eIF2α-P and eIF2α proteins (with a similar molecular weight), membranes were stripped off from the previously used antibody and probed again, according to standard protocols. Briefly, membranes were used immediately after chemiluminescence or as dried membranes that require an additional methanol activation. In either case, membranes were washed with bi-distilled water, incubated in a 250 mM solution of sodium hydroxide (NaOH), washed in bi-distilled water, and then in the respective buffer of the antibody to be used. After a blockage of 30 min in 5% (*w/v*) non-fat milk in 0.05% (*v/v*) Triton-TBS 1×, membranes are probed o/n, at 4 °C, with shacking, with rabbit anti-eIF2α (Cell Signaling; Danvers, MA, USA) diluted 1:1000 in 5% (*w/v*) non-fat milk in 0.05% (*v/v*) Triton-TBS 1×. Detection was performed as described above, followed by enhanced chemiluminescence.

### 2.6. RNA Extraction and cDNA Synthesis

Total RNA isolation was performed using TRIzol reagent (Invitrogen; Waltham, MA, USA) following the manufacturer’s protocol. Total RNA was then treated with RNase-free DNase I (Promega) for approximately 1 h at 37 °C, and the RNA was purified using phenol-chloroform, as described in standard protocols. cDNA was synthesized using the NZY Reverse Transcriptase (NZYTech) and oligo(dT) primers (NZYTech), as instructed by the manufacturer.

### 2.7. RT-qPCR

The cDNA synthesized was used as a template for the quantification of the mRNA expression levels of *ABCE1* (primers #5 and #6 in Table S3) and glyceraldehyde-3-phosphate dehydrogenase (*GAPDH*; primers #7 and #8 in Table S3) by quantitative PCR (qPCR) using the SYBR^®^ Green PCR Master Mix (Applied Biosystems; Waltham, MA, USA), as instructed by the manufacturer, in an Applied Biosystems^®^ 7500 Real-time PCR System (Applied Biosystems). The amplification program was the following: initial denaturation at 95 °C for 10 min, followed by 40 cycles of 95 °C for 15 s and 60 °C for 30 s. The mRNA levels of *ABCE1* were normalized to the mRNA levels of the internal control, GAPDH, and the relative mRNA expression levels were calculated by applying the comparative Ct method (ΔΔCt). For that, amplification efficiencies were calculated for each set of primers using serial dilutions of a cDNA sample.

### 2.8. Semi-Quantitative RT-PCR

The cDNA synthesized was used as a template for the quantification of the mRNA expression levels of *FLuc* (primers #1 and #2 in Table S3) and *RLuc* (primers #3 and #4 in Table S3) by semi-quantitative PCR using the GoTaq^®^ DNA polymerase (Promega) following the manufacturer’s protocol. The thermal cycling conditions were the following: initial denaturation at 95 °C for 4 min; 30 cycles of 95 °C for 30 s (denaturation), 55 °C for 30 s (annealing), and 72 °C for 1 min (extension); and then a final extension at 72 °C for 10 min. After amplification, 15 µL of the PCR products were resolved by electrophoresis in a 3% (*w/v*) agarose gel stained with ethidium bromide. The density of the bands was quantified using ImageJ software (version 1.52n; Bethesda, MD, USA), and the absolute mRNA amount was determined using a standard calibration curve performed with serial dilutions of a cDNA sample. Luciferase mRNA levels of each condition are obtained by normalizing FLuc to RLuc mRNA levels.

### 2.9. Statistical Analysis

The experimental analyses were performed at a minimum number of three independent assays, and the results are expressed as mean ± standard error of the mean (SEM). Student’s t-test, two-tailed and unpaired, was applied for statistical significance: *p* < 0.05 (*), *p* < 0.01 (**) and *p* < 0.001 (***).

## 3. Results

### 3.1. The Human ABCE1 5′-Leader Sequence Contains Five AUG and Five Non-AUG uORFs

**Figure 1:**
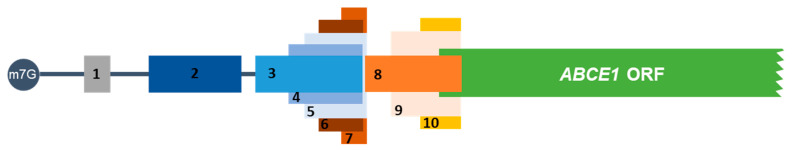
The human *ABCE1* 5′-leader sequence contains five AUG and five non-AUG uORFs. Schematic representation of the five AUG and five non-AUG uORFs in the 5′-leader sequence of the human *ABCE1* mRNA.

The human *ABCE1* mRNA 5′-leader sequence is a 515 nt-long sequence, as annotated in the Ensembl database (assembly GRCh37) with the transcript ID ENST00000296577. Our in silico analysis, using the ExPASy Translate tool, showed five AUG start codons in frame with stop codons (UGA) located within the *ABCE1* 5′-leader sequence, constituting five AUG uORFs (uORF1-5; Figure 1 and Table 1). Accordingly, Vanderperre and co-workers predicted bioinformatically the presence of two AUG uORFs within the *ABCE1* 5′-leader sequence [63], those corresponding to uORF2 and uORF3 identified by us using ExPASy. The first AUG uORF (uORF1) is the smallest of the five AUG uORFs, with only 36 nts, and is the closest to the 5′-end of the transcript with an intercistronic distance of 426 nts relative to the main coding sequence. The uORF2 has a 132 nts-long sequence and an intercistronic distance of 252 nts relative to the main ORF. The uORF3 and uORF4 with, respectively, 144 and 96 nts of length share the same stop codon located 94 nts upstream of the main AUG. The last AUG uORF, uORF5, is a 78 nt-long sequence and presents an intercistronic distance of 84 nts relative to the main AUG; uORF5 overlaps its first 71 nts with uORF3 and uORF4 (Figure 1). None of the five AUG uORFs has the initiation codon in an optimal Kozak consensus context [65] (Table 1), conversely to the AUG of the *ABCE1* main ORF that is in a strong Kozak context with -3 and +4 positions occupied by a G nucleotide. From the five uORFs, only uORF1 and uORF5 have a suitable Kozak context: uORF1 has an A at position -3, and uORF5 has a G at position +4. Additionally, uORF1 also has a G at position -6 that is described to improve translation initiation efficiency [65,66].

In the human *ABCE1* mRNA 5′-leader sequence, there are also five uORFs with non-canonical start codons (uORF6-10; Figure 1 and Table 1), as identified by a Ribo-Seq study performed in HCT116 cells [61]. The first non-AUG uORF, called uORF6 (since this uORF initiates downstream of uORF5 start codon), is a 60 nt-long uORF, has a GUG start codon, and is in frame with the third and fourth AUG uORFs (uORF3 and uORF4), sharing the same stop codon (Figure 1). The uORF7, which is the smallest non-AUG uORF, encompassing 36 nts, also starts with a GUG codon and shares the stop codon with uORF5 (Figure 1). Regarding the remaining non-AUG uORFs (uORF8-10), they share the same stop codon located 41 nts downstream of the main AUG, creating three overlapped uORFs (Figure 1). uORF8 is 129 nt-long, and its GUG start codon is located 88 nts upstream of the main AUG. uORF9 and uORF10 have 102 and 63 nts in length, respectively. Additionally, uORF9 starts with a GUG codon that is located 61 nts upstream of the main AUG, and uORF10 has a CUG initiation codon at 22 nts upstream of the main start codon. None of the five non-AUG initiation codons is in an optimal sequence context [30,67] (Table 1). Three of them have one nucleotide in a suitable position: uORF7 and uORF10 have a G in position +4, and uORF8 presents an A at position −3. Additionally, uORF6, uORF7, and uORF8 have a U at position −6.

The high complexity of the uORF organization within the 5′-leader sequence of the human *ABCE1* mRNA raises the question about the function of these uORFs in the translational regulation of the main coding sequence.

### 3.2. The ABCE1 5′-Leader Sequence Has Translation-Inhibitory AUG uORFs

**Figure 2:**
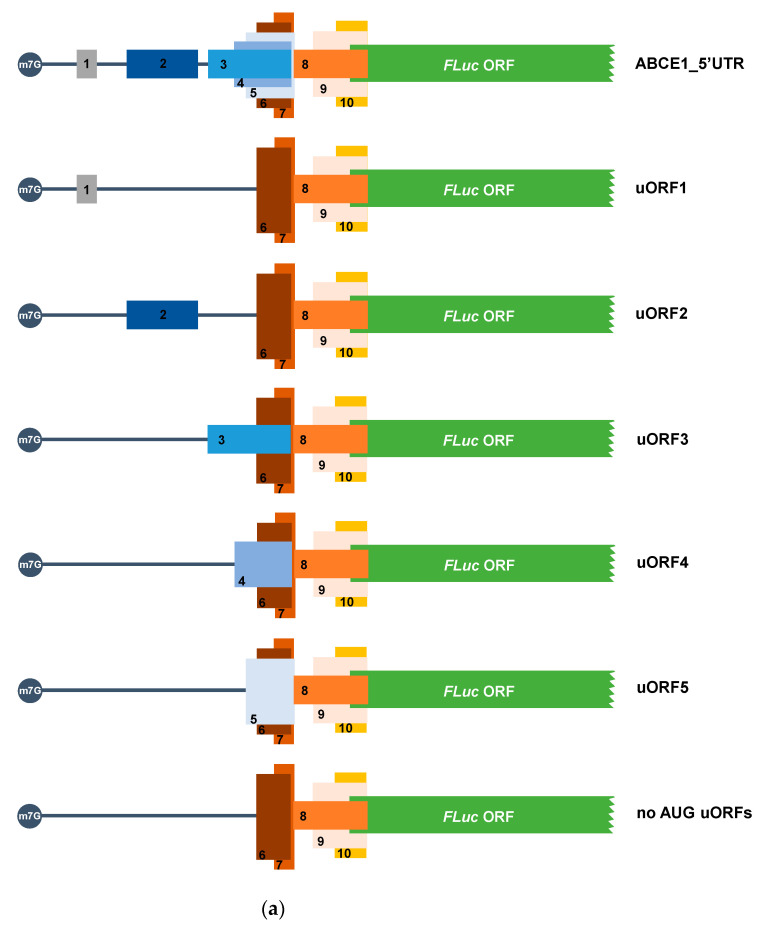

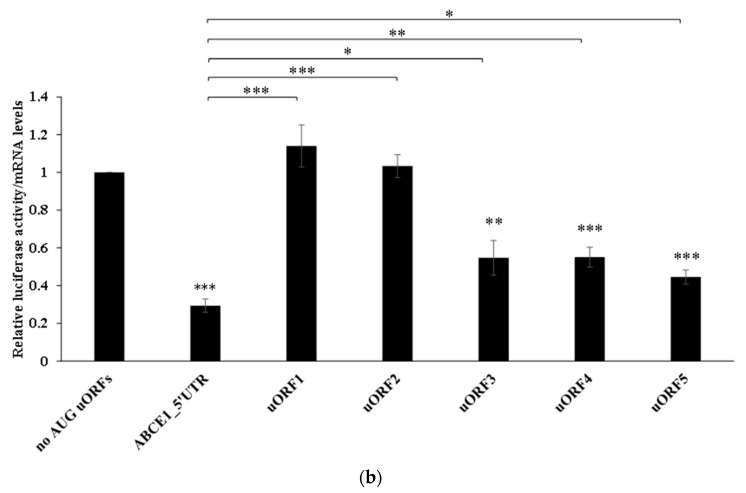
In the native configuration, the *ABCE1* AUG uORFs significantly repress translation of the downstream main ORF, but when isolated, only uORF3, uORF4, and uORF5 function as repressors. (**a**) Schematic representation of the constructs used to study the translational regulatory function of the *ABCE1* AUG uORFs. The human *ABCE1* 5′-leader sequence was cloned into the pGL2-FLuc vector upstream of the FLuc ORF (green box), obtaining the ABCE1_5′UTR construct. By site-directed mutagenesis, and using the ABCE1_5′UTR vector as a template, each AUG initiation codon was mutated (ATG→TTG), creating the constructs with only one functional AUG uORF (uORF1-5, represented here by small boxes and only by its uORF number, 1 to 5, to simplify the scheme) or without functional AUG uORFs (“no AUG uORFs”). Non-AUG uORFs (represented in small boxes with the numbers 6 to 10) are maintained intact in these constructs. (**b**) HCT116 cells were transiently co-transfected with each one of the constructs described in (**a**), and the pRL-TK plasmid expressing RLuc, used as a transfection efficiency control. Twenty-four hours post-transfection, cells were harvested and lysed. Luciferase activity was measured by luminometry assays. The results are expressed as relative luciferase activity determined by normalizing FLuc activity to that of RLuc. Luciferase mRNA levels were determined by semi-quantitative RT-PCR, and relative luciferase mRNA levels were determined by normalizing FLuc mRNA levels to that of RLuc for each transfected condition. Then, relative luciferase levels were normalized to the corresponding relative mRNA levels, and these values were compared to the one of “no AUG uORFs”, arbitrarily defined as 1. The results are presented as mean ± SEM from at least three independent experiments. Student’s t-test was applied for statistical significance: *p* < 0.05 (*), *p* < 0.01 (**), and *p* < 0.001 (***).

To analyze if the predicted AUG uORFs regulate translation of *ABCE1* transcript, we designed an expression vector in which the 515 nt-long 5′-leader sequence of the human *ABCE1* mRNA was cloned upstream of the FLuc coding sequence, driven by the hCMV promoter, in the pGL2-enhancer vector (called ABCE1_5′UTR) (Figure 2a). Next, we mutated all five ATGs to TTGs, generating a construct in which no functional AUG uORFs exist (called “no AUGs uORFs”) (Figure 2a). In addition, to determine which uORF bears translation repressive properties, each AUG start codon was altered separately to TTG, thus obtaining constructs with only one functional AUG uORF (called uORF1, uORF2, uORF3, uORF4, and uORF5; Figure 2a). HCT116 cells were transiently co-transfected with each one of the above-mentioned constructs and with the reporter vector expressing RLuc, the pRL-TK. Twenty-four hours post-transfection, cell extracts were obtained and analyzed by luminometry assays. The relative luciferase activity was quantified by normalizing FLuc activity to that of the RLuc and then by normalizing the FLuc/RLuc ratio from each transfected condition to the one of the “no AUG uORFs”, arbitrarily defined as 1. In addition, the luciferase mRNA levels were quantified by semi-quantitative RT-PCR. Relative mRNA levels were determined by normalizing FLuc mRNA levels to those of RLuc for each construct, followed by normalization to the results of the “no AUG uORFs”, arbitrarily defined as 1. For each case, levels of relative luciferase were normalized to the corresponding relative mRNA levels.

Expression of the ABCE1_5′UTR construct into HCT116 cells induced a decrease in relative luciferase activity, representing a significant ~3.4-fold repression when compared to that of the “no AUG uORFs” (Figure 2b). This result shows that the AUG uORFs at the *ABCE1* mRNA 5′-leader sequence are able to repress the translation of the main coding sequence. Regarding the ability of each of the AUG uORFs to repress translation, we observed that uORF1 or uORF2 do not affect the relative luciferase activity levels when comparing to the results obtained from the “no AUG uORFs” construct (Figure 2b), which is indicative of a lack of repressive activity by these two AUG uORFs. Conversely, uORF3, uORF4, and uORF5 induce a decrease in relative luciferase activity (Figure 2b); indeed, uORF3 and uORF4 contribute to a repression of ~1.8-fold, whereas uORF5 inhibits around 2.2-fold the relative luciferase activity, making uORF5 the most repressive AUG uORF. Of note, none of the constructs carrying only one of the repressive AUG uORFs exhibit a repressive effect as that of the intact 5′-leader sequence in the construct ABCE1_5′UTR. Instead, we observed an increase in the relative luciferase activity of ~1.9-fold for uORF3 and uORF4 and ~1.5-fold for uORF5, relative to the luciferase expression induced by the ABCE1_5′UTR construct. This result points out a synergistic/additive effect and a fail-safe mechanism of these three AUG uORFs in the repression of the main ORF.

**Figure 3:**
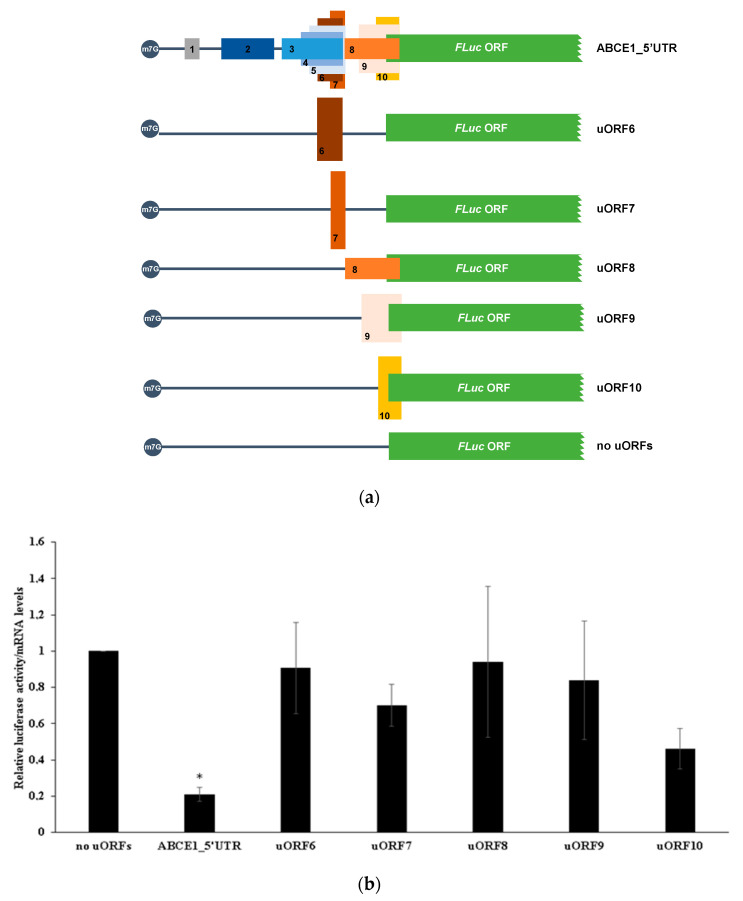
The non-AUG uORFs within the *ABCE1* mRNA 5′-leader sequence do not seem to significantly repress translation of the main ORF. (**a**) Schematic representation of the constructs used to study the translational regulatory function of the *ABCE1* non-AUG uORFs. The ABCE1_5′UTR, containing the intact *ABCE1* 5′-leader sequence, was already described in Figure 2a. By site-directed mutagenesis and using the “no AUG uORFs” construct as a template, each non-AUG initiation codon was mutated (CTG/GTG→GCG), creating the constructs with only one functional non-AUG uORF (uORF6-10, represented here by small boxes and only by its uORF number, 6 to 10, to simplify the scheme) or without any uORF (called “no uORFs”). (**b**) Expression of each construct was analyzed as described in Figure 2b legend, but using “no uORFs” construct expression data for the comparison analysis. The results are presented as mean ± SEM from at least three independent experiments. Student’s t-test was applied for statistical significance: *p* < 0.05 (*).

To test if similar regulation could be attributed to the non-AUG uORFs present in the 5′-leader region of the *ABCE1* transcript, site-directed mutagenesis was performed in each non-AUG start codon (CTG/GTG→GCG) using the “no AUG uORFs” construct as a template, obtaining constructs with no functional uORFs (without AUG nor non-AUG start codons, called “no uORFs” construct), and constructs carrying only one functional non-AUG uORF (called uORF6, uORF7, uORF8, uORF9, and uORF10) (Figure 3a). The expression of these constructs was analyzed as the previous ones. A significant decrease in the relative luciferase activity of the ABCE1_5′UTR construct was observed when compared to that of the “no uORFs” construct, representing a ~4.8-fold repression (Figure 3b). Nevertheless, none of the non-AUG uORFs has a significant inhibitory effect on the expression of the main ORF (Figure 3b). This result indicates that the *ABCE1* non-AUG uORFs do not seem to significantly contribute to the translational regulation of its main ORF.

**Figure 4:**
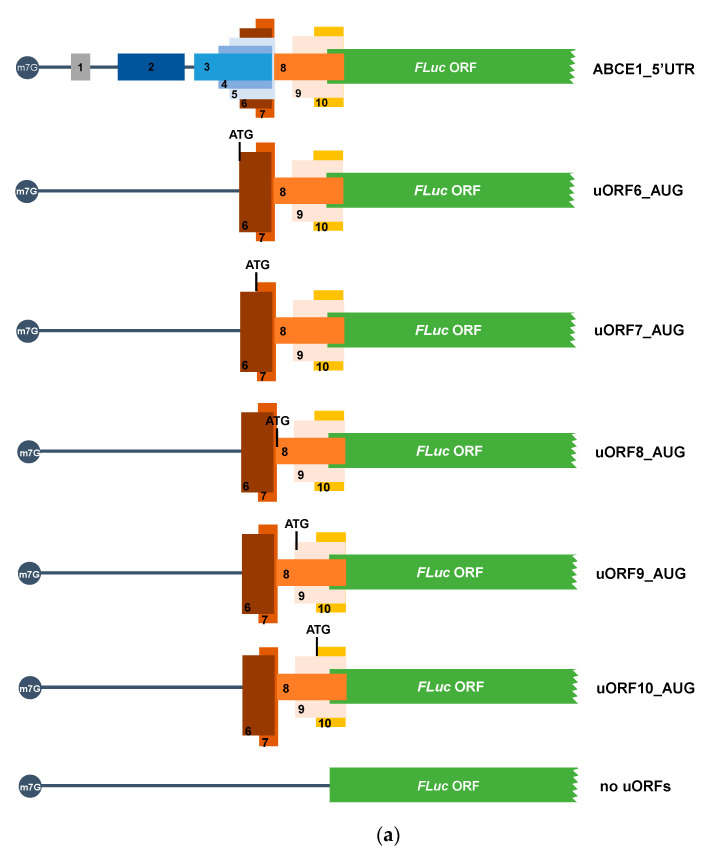

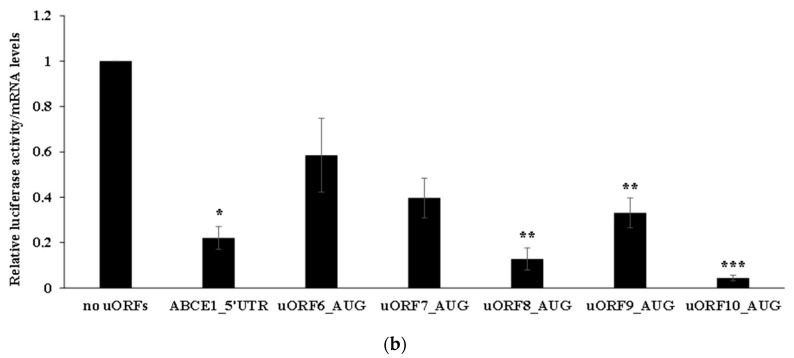
Mutating the *ABCE1* non-AUG to AUG uORFs increases their repressive effect over the main coding sequence. (**a**) Schematic representation of the constructs used. The ABCE1_5′UTR construct, containing the intact *ABCE1* 5′-leader sequence, and the “no uORFs” construct, without any functional AUG and non-AUG uORFs, were already described in Figure 2a and Figure 3a, respectively. By site-directed mutagenesis, and using the construct “no AUG uORFs” (described in Figure 2a) as a template, the start codon of each one of the non-AUG uORFs was mutated (CTG/GTG→ATG), and the following constructs were obtained: uORF6_AUG, uORF7_AUG, uORF8_AUG, uORF9_AUG, and uORF10_AUG. (**b**) Expression of each construct was analyzed as described in Figure 2b legend, but using “no uORFs” construct expression data for the comparison analysis. The results are presented as mean ± SEM from at least three independent experiments. Student’s t-test was applied for statistical significance: *p* < 0.05 (*), *p* < 0.01 (**) and *p* < 0.001 (***).

We next examined if the lack of inhibitory activity observed for the *ABCE1* non-AUG uORFs is due to their frequent ribosomal bypass as a consequence of the non-canonical nature of their start codons. For that, the start codon of each of the non-AUG uORFs was mutated to an AUG (CTG/GTG→ATG), using as template the construct “no AUG uORFs”, obtaining the constructs named uORF6_AUG, uORF7_AUG, uORF8_AUG, uORF9_AUG, and uORF10_AUG (Figure 4a). In each construct, only one of the non-AUG start codons was mutated, and all constructs contain the wild-type sequence context. Their expression in HCT116 cells (Figure 4b) shows that when the non-canonical initiation codon is replaced by the canonical AUG start codon, there is repression of the relative luciferase activity in all transfected constructs when compared to that of the “no uORFs”. Of note, repression exerted by uORF8, uORF9, and uORF10 becomes very significant (Figure 4b). This indicates that the uORF start codons become much more recognized by the scanning ribosomes.

Together, these results indicate that the native non-canonical start codons of these five uORFs are mainly bypassed by the scanning ribosomes, allowing translation of the main ORF and exerting no significant translational repression (Figure 3b).

In summary, the uORFs presented in the 5′-leader sequence of the human *ABCE1* transcript regulate expression of the main coding sequence in HCT116 cells, and this seems to be fully accomplished by the AUG uORFs, especially by uORF3, uORF4, and uORF5, which may function in a fail-safe manner to maximize translational inhibition of the main ORF.

### 3.3. The ABCE1 AUG uORFs Have the Potential to Be Translated

**Figure 5:**
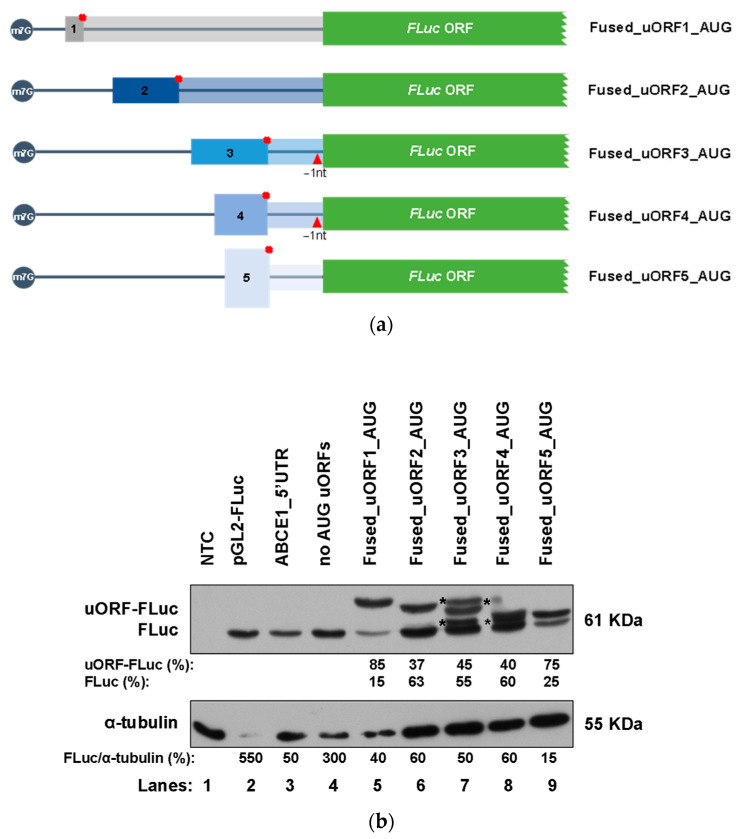
The five *ABCE1* AUG uORFs have the potential to be translated, being uORF1 and uORF5 the most efficiently recognized by the ribosomes. (**a**) Schematic representation of the constructs, in which the AUG uORF was fused in frame with the FLuc coding sequence. In the constructs uORF3 and uORF4, 1 nucleotide (T) was deleted from the intercistronic sequence, and, in uORF1-5, the in-frame stop codon was mutated (TGA→GGA), resulting in the following constructs: Fused_uORF1_AUG, Fused_uORF2_AUG, Fused_uORF3_AUG, Fused_uORF4_AUG, and Fused_uORF5_AUG. The red cross represents the point mutation at the uORF’s stop codons, and the red triangle indicates the 1-nucleotide deletion. The five non-AUG uORFs are present in these constructs but not represented in the figure to simplify the scheme. (**b**) HCT116 cells were transiently transfected with each one of the constructs depicted in (**a**) or, ABCE1_5′UTR, “no AUG uORFs” (both described in Figure 2a), or the pGL2-FLuc empty vector (to monitor native FLuc expression). Native FLuc protein and N-extended uORF-FLuc isoforms were assessed by Western blot using a specific antibody against FLuc. Alpha-tubulin was used as a loading control. This is a representative immunoblot from at least three independent experiments. The asterisks (*) represent translation initiation at other initiators; NTC: non-transfected control.

To verify that the AUG uORFs have the potential to be recognized by the scanning ribosomes and actively translated, we next constructed reporter vectors carrying each one of the AUG uORFs fused in frame with the FLuc coding sequence. The resulting constructs were named: Fused_uORF1_AUG, Fused_uORF2_AUG, Fused_uORF3_AUG, Fused_uORF4_AUG, and Fused_uORF5_AUG (Figure 5a). Since the uORF’s start codons are in frame with the main coding sequence, inactivation of the respective uORF stop codons was predicted to result in the production of an elongated FLuc protein. To confirm this, these constructs were expressed in HCT116 cells as before, and production of FLuc protein and N-extended uORF-FLuc isoforms were assessed by Western blot (Figure 5b).

Results show that the ABCE1_5′UTR and “no AUG uORFs” constructs (lanes 3 and 4, respectively, Figure 5b) express a protein with a molecular weight of 61 kDa, corresponding to the native FLuc protein that is also observed in cells transfected with the empty vector (pGL2-FLuc; lane 2, Figure 5b). Of note, a decrease in FLuc expression from the ABCE1_5′UTR construct (lane 3, Figure 5b) is observed in comparison to the expression of FLuc from the “no AUG uORFs” construct (lane 4, Figure 5b). This result is in agreement with a repression activity promoted by the intact *ABCE1* mRNA 5′-leader sequence, as previously shown (Figure 2b). All fused constructs (lanes 5–9, Figure 5b) express proteins with a molecular weight higher than the one expected for the native FLuc protein, corresponding to the uORF-FLuc fused protein. These results demonstrate that the uAUGs are, in fact, recognized by the scanning ribosomes, and thus, all the AUG uORFs have the potential to be translated. Additionally, all the fused constructs also express the native FLuc protein (Figure 5b), indicating some degree of ribosomal bypass of the five AUG uORFs. Indeed, for uORF2, uORF3 and uORF4 fused constructs, as shown in lanes 6 to 8 of Figure 5b, there is a higher expression of the native FLuc protein than of the corresponding uORF-FLuc fused isoforms. In the case of uORF1 and uORF5, this bypass is less efficient, as demonstrated by a lower expression of the native FLuc protein compared to that of the uORF-FLuc fused protein (lanes 5 and 9, respectively, Figure 5b), indicating a more efficient recognition of the corresponding uAUGs. As above referred, none of the five AUG uORFs has the initiation codon in an optimal Kozak consensus context (Table 1), which explains the expression of both uORF-FLuc and FLuc proteins in all cases. Furthermore, the fact that only uORF1 and uORF5 have their initiators in a suitable Kozak context explains their higher expression of uORF-FLuc relative to FLuc protein, contrary to what occurs in the other cases, where the Kozak consensus sequence is weaker (lanes 5 and 9, versus lanes 6, 7, and 8, Figure 5b). In parallel, we also observe additional bands in lanes 7 and 8 (labeled with asterisks; Figure 5b) that may correspond to N-terminally extended FLuc proteins due to translation initiation at other in-frame start codons.

**Figure 6:**
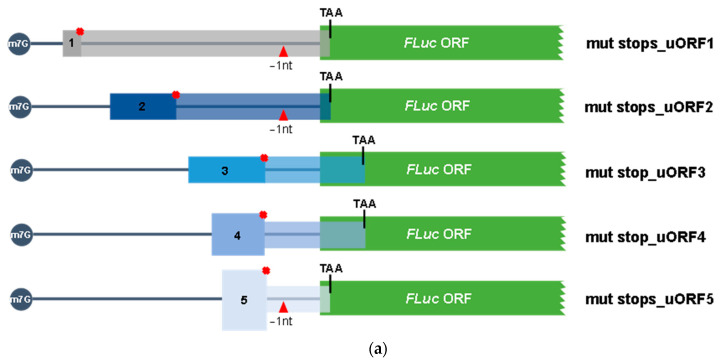

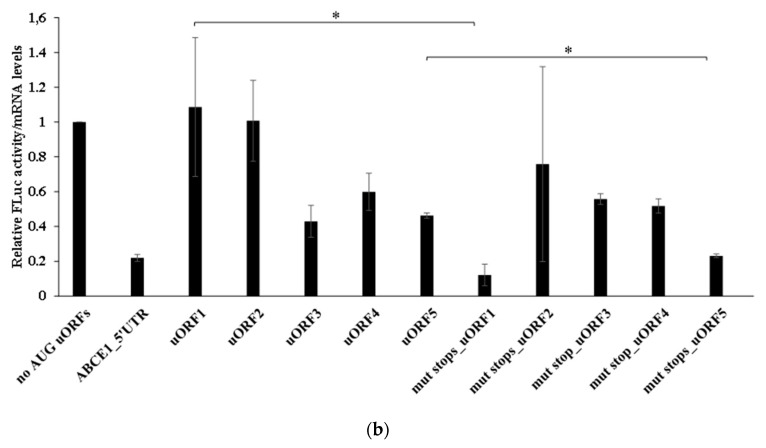
uORF 2, uRF3, and uORF4 present in the *ABCE1* 5′-leader sequence have the potential to be frequently bypassed by the ribosomes. (**a**) Schematic representation of the constructs obtained by series of sequential mutagenesis to obtain out-of-frame and overlapped uORFs with the main coding sequence. In the uORF1, uORF2 and uORF5 constructs, 1 nucleotide (T) was deleted in the intercistronic space and, for all the five AUG uORF-individual constructs (uORF1-5), each in-frame stop codon was mutated (TGA→GGA), resulting in the constructs “mut stops_uORF1”, “mut stops_uORF2”, “mut stop_uORF3”, “mut stop_uORF4”, and “mut stops_uORF5”. The red cross represents the point mutations at the uORF’s stop codons, and the red triangle indicates the 1-nucleotide deletion. The five non-AUG uORFs are present in these constructs but not represented in the figure to simplify the scheme. (**b**) HCT116 cells were transiently co-transfected with each one of the constructs described in (**a**), or ABCE1_5′UTR, “no AUG uORFs”, or the constructs with only one functional AUG uORF (uORF1-5) already described in Figure 2a, and the pRL-TK plasmid. Expression of these constructs was analyzed as described in Figure 2b legend. The results are presented as mean ± SEM from at least three independent experiments. Student’s t-test was applied for statistical significance: *p* < 0.05 (*).

To unequivocally appreciate how much each uORF can be recognized or bypassed by the translating ribosomes, we analyzed constructs carrying each one of the AUG uORFs out-of-frame and overlapped with the main coding sequence (constructs called “mut stops_uORF1”, “mut stops_uORF2”, “mut stop_uORF3, “mut stop_uORF4”, “mut stops_uORF5”; Figure 6a). Thus, if the uORF initiation codon is bypassed, it is expected for the scanning ribosomes to succeed in recognizing the main ORF’s initiation codon and initiating translation, while translation reinitiation is completely abolished. To perform this analysis, HCT116 cells were transiently transfected with each one of these constructs, or with the constructs carrying only one functional AUG uORF, the ABCE1_5′UTR, or the “no AUG uORFs”, and their relative luciferase activity compared to that of the corresponding construct with only one functional AUG uORF (Figure 6b). The “mut stops_uORF1” construct induced very low levels of relative luciferase activity when compared to the expression of uORF1 (Figure 6b).

This result demonstrates that native uORF1 is not significantly bypassed, but instead, the initiation codon of uORF1 is efficiently recognized by the scanning ribosomes, leading to an efficient translation of uORF1. This result is in agreement with the possible occurrence of translation reinitiation at the main ORF after uORF1 translation. This corroborates the nature of uORF1 as a ribosomal barrier. In addition, the “mut stops_uORF5” induces a lower relative luciferase activity (~2-fold decrease) when comparing to uORF5 expression levels (Figure 6b), indicating that half of the ribosomes can bypass uORF5 start codon and initiate translation at the FLuc AUG. On the other hand, “mut stops_uORF2” expresses FLuc at a level not significantly different from that of uORF2 (Figure 6b). Thus, the uORF2 initiation codon is bypassed by the scanning ribosomes that efficiently initiate translation at the main AUG. This result is also in accordance with data from Figure 5b, where we observed that the Fused_uORF2_AUG expresses the uORF-FLuc fusion protein at a lower level than the native FLuc protein (lane 6, Figure 5b). In what concerns the “mut stop_uORF3” and “mut stop_uORF4” constructs, we observe that their relative luciferase activities are not significantly different from those of uORF3 and uORF4, respectively (Figure 6b), highlighting that uORF3 and uORF4 can be significantly bypassed by the scanning ribosomes. Overall, these results support those from Figure 5b.

**Figure 7:**
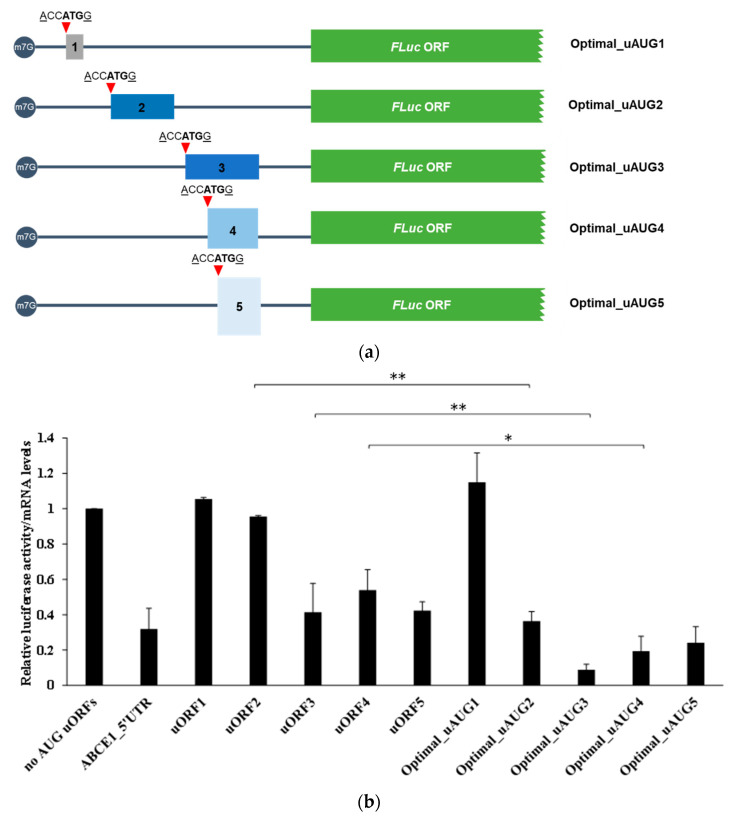
uORF1 and uORF5 present in the *ABCE1* 5′-leader sequence have the potential to be efficiently recognized and translated by the ribosomes, allowing translation reinitiation to occur at the main AUG. (**a**) Schematic representation of the constructs where the Kozak sequence context of each uORF initiation codon was replaced by an optimal Kozak sequence context (ACCATGG; indicated by the red triangle), obtaining the constructs Optimal_uAUG1, Optimal_uAUG2, Optimal_uAUG3, Optimal_uAUG4, and Optimal_uAUG5. The five non-AUG uORFs are present in these constructs but not represented in the figure to simplify the scheme. (**b**) HCT116 cells were transiently co-transfected with each one of the constructs described in (**a**), or ABCE1_5′UTR, “no AUG uORFs”, or the constructs with only one functional AUG uORF (uORF1-5) already described in Figure 2a, and the pRL-TK plasmid. Expression of these constructs was analyzed as described in Figure 2b legend. The results are presented as mean ± SEM from at least three independent experiments. Student’s t-test was applied for statistical significance: *p* < 0.05 (*) and *p* < 0.01 (**).

To investigate the potential of translation reinitiation at the main ORF after translation of each one of the AUG uORFs, the context of each uAUG was replaced by an optimal Kozak sequence context (ACCATGG [65]), obtaining the constructs named Optimal_uAUG1, Optimal_uAUG2, Optimal_uAUG3, Optimal_uAUG4 and Optimal_uAUG5 (Figure 7a). With this alteration, it is expected an increase in recognition of the uAUGs by the scanning ribosomes, with the consequent translation of the uORFs, and thus, FLuc expression will only occur by translation reinitiation. Expression of these constructs was compared to that of the corresponding construct with only one functional AUG uORF (Figure 7b). Results show that FLuc expression in the Optimal_uAUG1 and uORF1 constructs is similar (Figure 7b), indicating that the scanning ribosomes frequently recognize the uAUG1, translate uORF1, and reinitiate translation at the main AUG. This result is in accordance with the very low levels of luciferase expression from the “mut stops_uORF1” construct (Figure 6b). In addition, the Optimal_uAUG5 construct expressed FLuc at levels not significantly different from those of the uORF5 construct (Figure 7b), once more, demonstrating that translation reinitiation at the main AUG can occur after translation of uORF5. Conversely, FLuc expression obtained by Optimal_uAUG2 construct is about 37% of that from uORF2 (Figure 7b), indicating that the native uORF2 can be more frequently bypassed by the ribosome than recognized by it, which initiates translation at the main AUG. The same pattern of expression is observed for the Optimal_uAUG4 versus uORF4, suggesting that uAUG4 can often suffer leaky scanning (Figure 7b). On the other hand, the FLuc expression of the Optimal_uAUG3 construct is almost undetectable as in comparison to that of uORF3, indicating that it does not allow translation reinitiation at the main AUG (Figure 7b). This result corroborates the frequent bypass of the uORF3 initiation codon by the scanning ribosomes.

### 3.4. ABCE1 uORF3 and uORF5 Are Competent Translational Repressors, Greatly Regulated by uORF1 and to a Less Extent by uORF2

**Figure 8:**
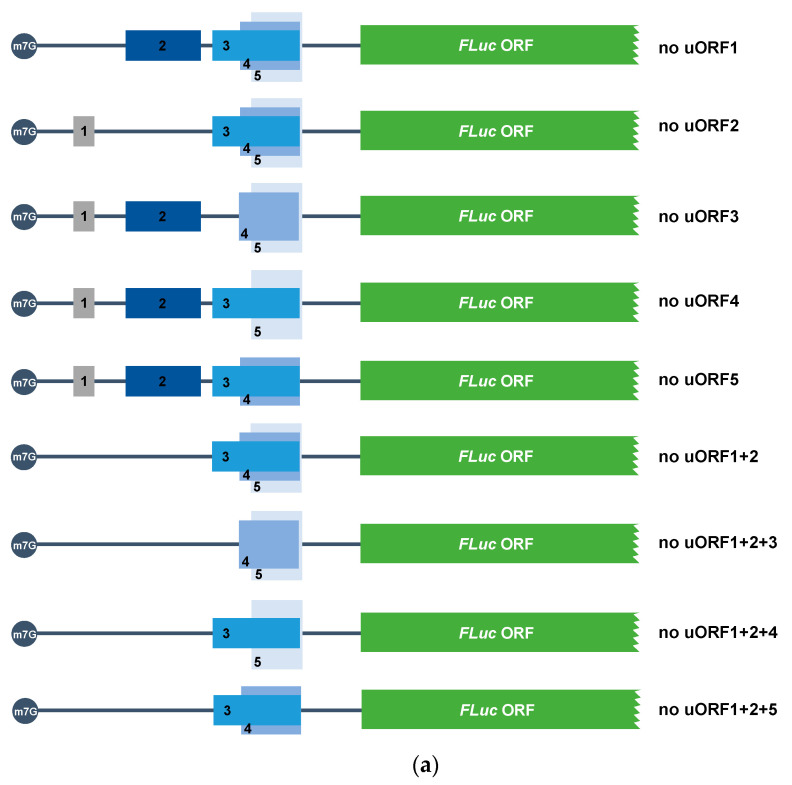

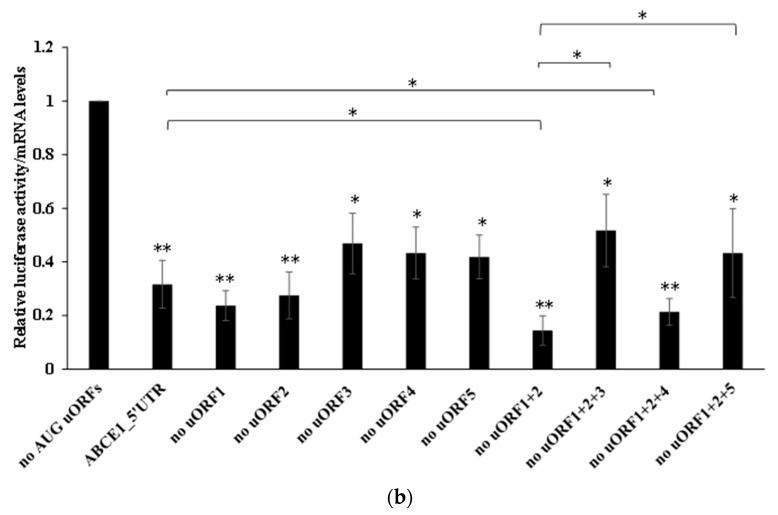
The *ABCE1* uORF1 and uORF2 regulate the level of repression exerted by uORF3, or uORF5. (**a**) Schematic representation of the constructs, in which only one uAUG codon is mutated (ATG→TTG), obtaining the constructs “no uORF1”, “no uORF2”, “no uORF3”, “no uORF4”, and “no uORF5”, or different combinations of mutated and functional uORFs, originating the constructs named “no uORF1+2”, “no uORF1+2+3”, “no uORF1+2+4”, and “no uORF1+2+5”. The five non-AUG uORFs are present in these constructs but not represented in the figure to simplify the scheme. (**b**) HCT116 cells were transiently co-transfected with each one of the constructs described in (**b**), or ABCE1_5′UTR, or “no AUG uORFs” (both described in Figure 2a), and the pRL-TK plasmid. Expression of these constructs was analyzed as described in Figure 2b legend. The results are presented as mean ± SEM from at least three independent experiments. Student’s t-test was applied for statistical significance: *p* < 0.05 (*), and *p* < 0.01 (**).

Taking into account the complexity of the 5′-leader sequence of *ABCE1* mRNA in terms of uORFs and knowing the potential of each AUG uORF to be translated or leaky scanned, we next decided to investigate how the five *ABCE1* AUG uORFs function when together in the native configuration, to regulate the expression of the main coding sequence. With this aim in mind, we mutated the *ABCE1* uAUGs in several possible combinations: (i) only one uAUG mutated (ATG → TTG), originating constructs named “no uORF1”, “no uORF2”, “no uORF3”, “no uORF4”, and “no uORF5”, and (ii) two or three uAUGs mutated, where uORF1 and uORF2 initiation codons are always mutated, originating the constructs named “no uORF1+2”, “no uORF1+2+3”, “no uORF1+2+4”, and “no uORF1+2+5” (Figure 8a). HCT116 cells were then transiently co-transfected with these constructs, and their expression was analyzed as before (Figure 8b).

In Figure 8b, we observe a general and significant decrease in relative luciferase activity of all the constructs analyzed, as compared to the expression levels of the “no AUG uORFs” control (without functional AUG uORFs). This points out that all the combinations of AUG uORFs have the power to regulate, to some extent, the translation of the main ORF. Comparing to the expression of the ABCE1_5′UTR construct, expression of “no uORF1”, “no uORF2”, “no uORF3”, “no uORF4”, and “no uORF5” is not significantly different (Figure 8b), indicating that the absence of one of these uORFs does not significantly compromise translational repression of the main ORF. These results are compatible with a mechanism in which *ABCE1* AUG uORFs function in a fail-safe manner to maintain strong translational repression of the main ORF.

Regarding the expression of “no uORF1+2”, a ~2.2-fold decrease in relative luciferase activity is observed relative to that of ABCE1_5′UTR (Figure 8b). Knowing that uORF1 is efficiently translated, and uORF2 is frequently leaky scanned (Figure 5b), these results indicate that uORF1 (and to a lesser extent, uORF2) might function as a barrier to ribosomal recognition of the downstream uORFs, allowing reinitiation to occur at the downstream main AUG. If uAUG1 and uAUG2 are not present, the ribosome will scan until it reaches a downstream uAUG. We hypothesize that without uORF1 and uORF2, more ribosomes can recognize the start codons of the inhibitory uORF3, uORF4, or uORF5. This will decrease the efficiency of translation reinitiation at the main AUG since, in this case, the intercistronic distance relative to the main ORF is shorter than from uORF1/uORF2. Thus, this will favor the repressive function of uORF3, uORF4, and uORF5 and explain the high level of translational repression observed in the “no uORF1+2” construct (Figure 8b). The relative luciferase activity observed for the constructs “no uORF1+2+3” and “no uORF1+2+5” is, respectively, ~3.6-fold and ~3.0-fold higher than the one of the construct “no uORF1+2” (Figure 8b). This significant increase in relative luciferase activity demonstrates once more that when one of the most inhibitory uORFs (uORF3 or uORF5) is eliminated, the other one compensates its function, maintaining the normal translational repression of the main ORF. On the contrary, the construct “no uORF1+2+4” is expressed at the same level as the “no uORF1+2” construct (Figure 8b), demonstrating that, in the native configuration, uORF4 does not significantly contribute to the repressive effect promoted by the AUG uORFs within the 5′-leader sequence of the *ABCE1* transcript.

Together, these results demonstrate that uORF1, and to a less extent uORF2, function as ribosomal barriers to the recognition of the uAUG3, or uAUG5, regulating the full inhibitory function that uORF3, or uORF5, can exert, and thus contributing to the maintenance of ABCE1 protein at physiological levels.

### 3.5. Although ABCE1 AUG uORFs Mediate Efficient Translational Repression during Thapsigargin-Induced ER Stress, Some Translational Derepression Occurs

**Figure 9:**
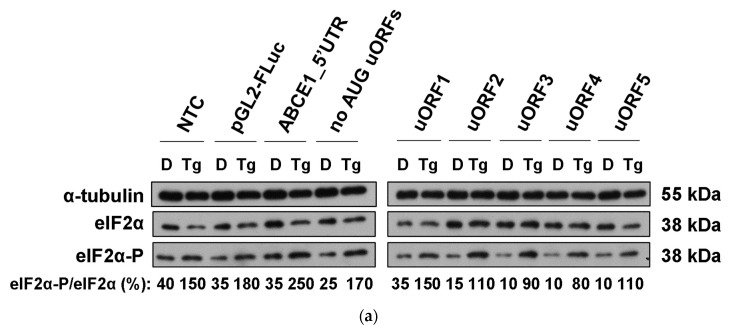

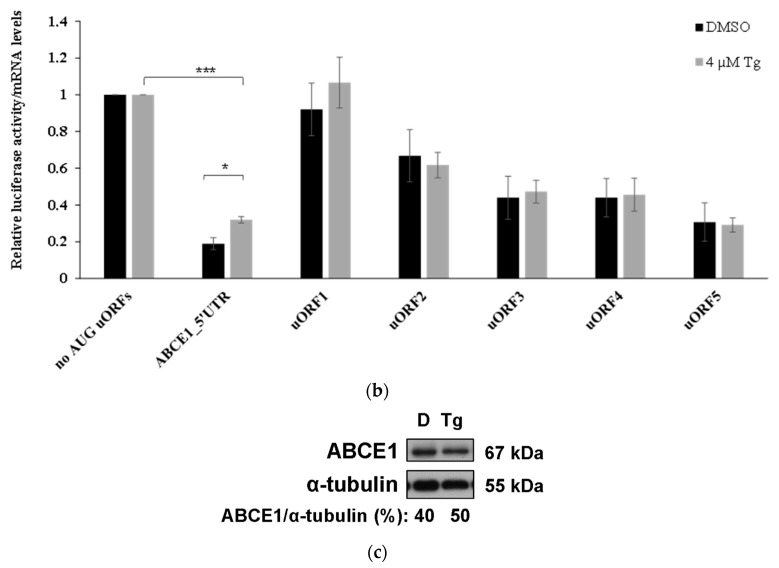
*ABCE1* AUG uORF-mediated translational repression is partially alleviated under ER stress induced by thapsigargin (Tg). HCT116 cells were transiently co-transfected with each one of the constructs described in Figure 2a and the pRL-TK plasmid. Four hours post-transfection, cells were treated with 4 µM of thapsigargin (Tg; gray bars) or the vehicle control of Tg (DMSO; black bars) for 24 h and then harvested and lysed. (**a**) The ER stress induced by Tg was assessed by Western blot using specific antibodies against eIF2α-P and the counterpart, eIF2α. Alpha-tubulin was used as a loading control. These are representative immunoblots from at least three independent experiments. NTC–non-transfected control. (**b**) Luciferase activity was assessed by luminometry assays (upper panel), and the corresponding luciferase mRNA levels by semi-quantitative RT-PCR (lower panel), and the relative expression was analyzed as described in Figure 2b legend. (**c**) The expression of endogenous ABCE1 protein was assessed in the absence (D, DMSO) or presence (Tg; tapsigargin) of ER stress induced by Tg, using specific antibodies against ABCE1. Alpha-tubulin was used as a loading control. The results are presented as mean ± SEM from at least three independent experiments. Student’s t-test was applied for statistical significance: *p* < 0.05 (*), and *p* < 0.001 (***).

A number of stresses, as in the case of ER stress, lead to a general shutdown and a quick reprogramming of protein synthesis, which include translation of selective subsets of mRNAs, such as those carrying uORFs [35,36,37]. Based on these data, we aimed to evaluate the impact of ER stress on the translational repression mediated by the *ABCE1* AUG uORFs. Therefore, HCT116 cells were transiently transfected with the constructs ABCE1_5′UTR, “no AUG uORFs”, or the reporter vectors carrying only one functional AUG uORF (uORF1-5), and cells were treated with thapsigargin to induce ER stress. The efficiency of the ER stress was monitored by immunoblotting of phosphorylated eIF2α protein levels (Figure 9a). Results show that ER stress did not significantly affect the inhibitory activity of the *ABCE1* AUG uORFs when each one was tested separately (Figure 9b). However, if in the native configuration, the *ABCE1* AUG uORFs become significantly less repressive (Figure 9b). In accordance, a small increase in endogenous ABCE1 protein expression was also observed upon induction of ER stress (Figure 9c). Thus, the translational regulation mediated by the five AUG uORFs within the 5′-leader sequence of *ABCE1* transcript responds to some extent to the environmental conditions.

### 3.6. The ABCE1 uORF-Mediated Translational Regulation Does Not Seem to Have an Oncogenic Role in Colorectal Cancer Cells

**Figure 10:**
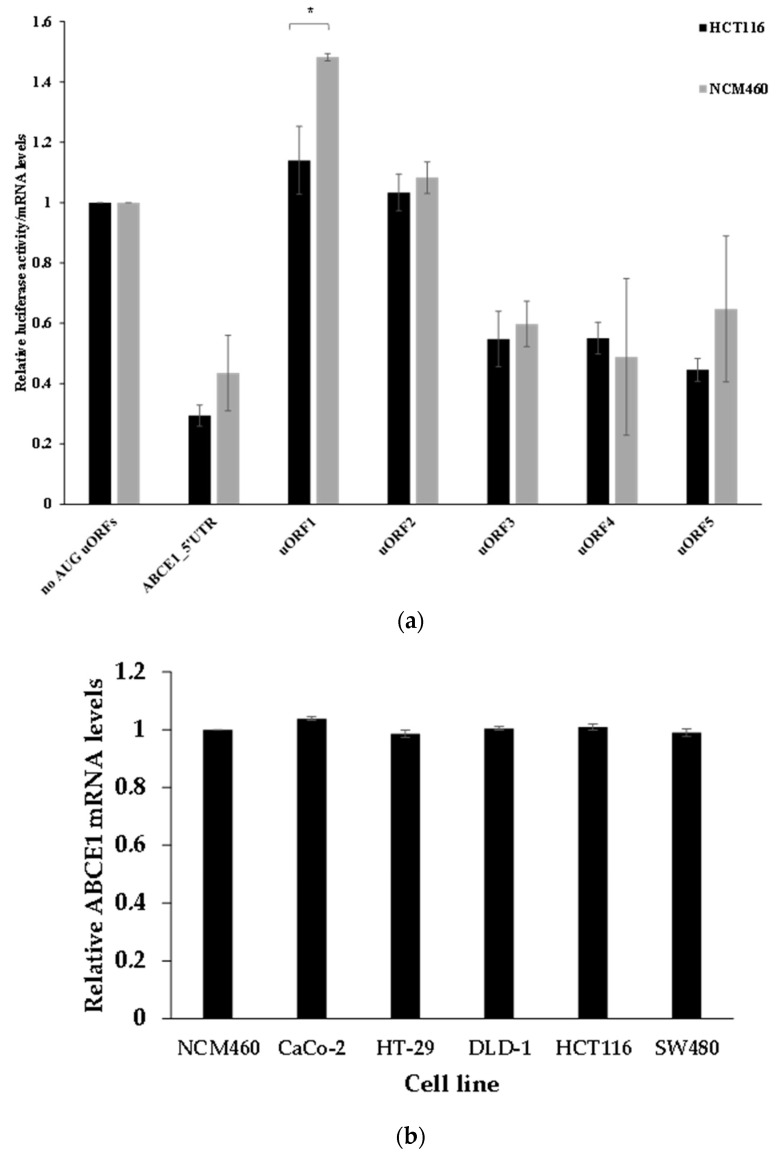

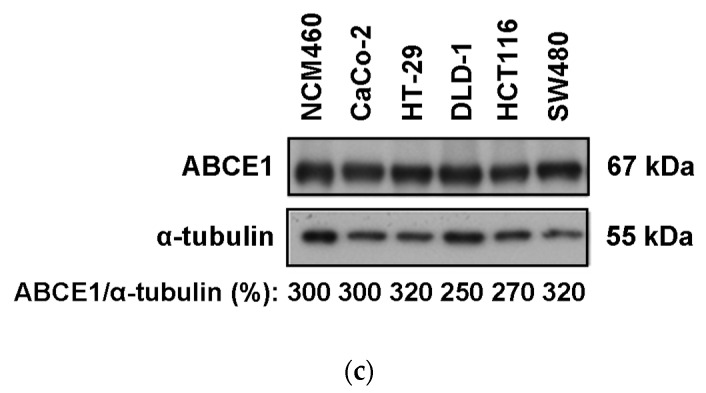
*ABCE1* AUG uORFs and ABCE1 protein do not have an oncogenic role. (**a**) NCM460 and HCT116 cells were transiently co-transfected with each one of the constructs described in Figure 2a, and their expression analyzed as described in Figure 2b legend. The data from HCT116 cells are the one displaying in Figure 2b. The results are presented as mean ± SEM from at least three independent experiments. Student’s t-test was applied for statistical significance: *p* < 0.05 (*). The *ABCE1* mRNA (**b**) and protein (**c**) levels were evaluated in the colorectal cancer cells lines CaCo-2, HT-29, DLD-1, HCT116, and SW480, as well as in the non-tumorigenic cell line NCM460. The *ABCE1* mRNA levels were assayed by RT-qPCR and normalized to the ones of the *GAPDH* internal control in each colorectal cell line; *ABCE1*/*GAPDH* ratio from each colorectal cancer cell line was compared to that of NCM460 cells. The results are presented as mean ± SEM from at least three independent experiments. The protein levels were obtained by Western blot using a specific antibody against ABCE1. Alpha-tubulin was used as a loading control. This is a representative immunoblot from at least three independent experiments.

Our results show that in the human colorectal carcinoma cell line HCT116, translation of the *ABCE1* mRNA is regulated by the AUG uORFs within its 5′-leader sequence (Figure 2b). This raises the question of if the *ABCE1* uORF-mediated translational regulation is dependent on the cancer cell environment and if it can be subject to change in non-tumorigenic cells, thus potentially being an important clue to understanding the transformation from non-neoplastic to cancerous colorectal cells. To address these questions, we studied the translational regulation mediated by the AUG uORFs of the *ABCE1* transcript in a non-tumorigenic versus colorectal cancer cell line. Accordingly, we analyzed the expression of the constructs ABCE1_5′UTR, “no AUG uORFs”, or the reporter vectors with only one functional AUG uORF (uORF1-5) (Figure 2a) in the non-neoplastic colorectal cell line NCM460, and results were compared to those obtained in HCT116 cells (Figure 2b). As shown in Figure 10a, in NCM460 cells, the ABCE1_5′UTR construct induces a ~2.3-fold repression in the relative luciferase activity when in comparison to the one obtained from the “no AUG uORFs” construct. This level of repression is comparable to the one already observed in HCT116 cells (Figure 2b and Figure 10a), indicating that the *ABCE1* AUG uORFs-mediated translational regulation is independent of the cell type. In addition, the translational regulation exerted by each of the five uORFs occurs with the same pattern in both cell lines (Figure 10a). These results reveal that the AUG uORFs of the *ABCE1* mRNA equally regulate translation in non-tumorigenic and in colorectal cancer cells, which is consistent with the lack of a role for these uORFs in the tumorigenic process of HCT116 cells. To better validate this observation, we compared the levels of *ABCE1* mRNA (Figure 10b) and protein (Figure 10c) in several colorectal cancer cells lines that are characteristic of different stages of colorectal cancer development (CaCo-2, HT-29, DLD-1, HCT116, and SW480) to the ones in a non-tumorigenic colorectal cell line (NCM460). As shown in Figure 10b,c, respectively, the *ABCE1* mRNA and protein levels are maintained unaffected in all the colorectal cancer cell lines tested and are at levels comparable to those in NCM460 cells, supporting the absence of a link between *ABCE1* uORF-mediated translational control and cancer progression. These results suggest that neither the process of *ABCE1* AUG uORFs-mediated translational control nor ABCE1 seems to have a direct role in the tumorigenic process of colorectal cancer.

## 4. Discussion

Human *ABCE1* 5′-leader sequence is extremely complex due to the presence of at least 10 uORFs (five starting with AUG and five with non-AUG initiation codons (Figure 1 and Table 1)) of unknown function. Here, we show that the uORFs present in the 5′-leader sequence of the human *ABCE1* transcript are able to efficiently repress translation of the main coding sequence in HCT116 colorectal cancer cells (Figure 2). Trying to dissect how each uORF functions in translational control, our data show that uORFs 3, 4, and 5 are able to downregulate the expression of the main coding sequence (Figure 2). On the contrary, the five non-AUG uORFs are devoid of regulatory function (Figure 3), which seems to be associated with the inefficient recognition of their initiation codons by the scanning ribosomes (Figure 4). In addition, we show that all the five *ABCE1* AUG uORFs have the potential to be translated, being uORF1 and uORF5 the most efficiently translated ones (Figure 5), while uORF2, uORF3, and uORF4 are frequently subjected to ribosomal leaky scanning (Figure 5 and Figure 6). Furthermore, uORF1 and uORF5 are permissive to the occurrence of efficient translation reinitiation at the main coding sequence (Figure 7). Nevertheless, when we look at these uORFs together in their native configuration, we observe that the complex uORF architecture in the 5′-leader sequence of the *ABCE1* mRNA is able to significantly repress expression of the downstream main coding sequence in about 70% in colorectal cancer cells (Figure 2 and Figure 3). This level of inhibition falls into the well-established range of 30–80% repression exerted by uORFs [9,68,69]. This repression is mainly achieved by two AUG uORFs, uORF3 and uORF5, and to a lesser extent, by uORF4 (Figure 2). Of note, as these uORFs are overlapped in the native configuration, they seem to function in a fail-safe mechanism. A similar mechanism was demonstrated for the two AUG uORFs of the human hemojuvelin mRNA: when uORF1, the strongest inhibitory uORF, is bypassed, it seems that the scanning ribosomes initiate translation at uORF2, maintaining main ORF expression at reduced levels [70].

The inhibitory activity of a uORF is positively correlated with (i) a strong uORF initiation codon Kozak context, (ii) a long distance from the transcript’s 5′-end to the uORF initiation codon, (iii) the presence of other uORFs (additive effect), (iv) a long uORF length, and/or (v) a short intercistronic distance [9,10,13,22,23]. Indeed, the repressive effect of uORF3, uORF4, and uORF5 can be associated with their long length, the short intercistronic distance, and/or the fact that their initiation codons are located far from the 5′-end of the transcript (Figure 1 and Table 1). However, only uORF5 is in a suitable Kozak sequence context (Table 1), which explains its higher translation efficiency (Figure 5), and accordingly, uORF5 is the most repressive uORF (Figure 2). In what concerns uORF3, although it is longer than uORF5, its initiation codon is in a suboptimal Kozak context (Table 1), which makes uORF3 to be more leaky scanned than uORF5 (Figure 6). On the other hand, uORF1 and uORF2 present the following features: (i) a suboptimal Kozak consensus sequence (although uORF1 has a better Kozak context than uORF2), (ii) their AUGs are closer to the 5′-end of the mRNA (especially for uORF1), and (iii) there is a long intercistronic distance relative to the main AUG (Figure 1 and Table 1). Additionally, uORF1 is the shortest AUG uORF (only 36 nts) in the *ABCE1* 5′-leader sequence (Table 1). Altogether, these characteristics demonstrate why both uORF1 and uORF2 are devoid of inhibitory activity. Furthermore, the slightly better Kozak context of uORF1 makes it more translatable than uORF2 (Figure 5). Thus, it seems that after translation of uORF1, or, less frequently, uORF2, uORF3, uORF4, and uORF5 can be, at least sometimes, leaky scanned, and ribosomes resume scanning until the main AUG is encountered, where they reinitiate translation (Figure 6 and Figure 7). Indeed, leaky scanning of uORF3 and uORF4 well correlates with their weak Kozak sequence context (Table 1). However, taking into consideration the repressive behavior of uORF3-5, it is expected that the ribosomes tend to recognize them (Figure 5). Thus, in the native configuration of *ABCE1* 5′-leader sequence, we expect that after uORF3, uORF4, or uORF5 translation, different outcomes can occur, which reduce the number of ribosomes at the main AUG: (i) the ribosomes can be dissociated and recycled, or (ii) the ribosomes can be stalled during translation elongation or termination.

On the other hand, we observed that the non-AUG uORFs within the *ABCE1* 5′-leader sequence are deprived of significant repressive functions (Figure 3), which agrees with their non-optimal initiation codons (Figure 4). It was shown that in the glutamyl-propyl-tRNA synthase (*EPRS*) transcript, there are two non-AUG uORFs (CUG and UUG start codons in uORF1 and uORF2, respectively), which induce a downregulation of the main coding sequence expression if the initiation codons are mutated to an AUG in an optimal context [71]. A similar result was obtained by us when we mutated the non-AUG to AUG codons (Figure 4).

Our results support a model in which after *ABCE1* uORF1 translation, the ribosome will, at least some times, reinitiate translation at one of the subsequent start codons upstream of the main AUG, as for instance, the initiation codons of the repressive uORF3, uORF4, or uORF5. A similar mechanism was shown for the general control protein (*GCN4*) transcript that, in physiological conditions, its AUG uORF1 is responsible for translation reinitiation at the three downstream inhibitory AUG uORFs, leading to GCN4 downregulation [72,73]. However, ribosomes in the *ABCE1* transcript do not seem to act exactly in the same way, since abolishing only uORF1 translation results in a similar repression level to the one performed by all the AUG uORFs in the intact *ABCE1* 5′-leader sequence (Figure 8). Additionally, impairing uORF2 translation has the same outcome observed when the uORF1 is inactivated (Figure 8). On the other hand, our data also show that in the absence of uORF1 and uORF2, the repression exerted by uORF3, uORF4, and uORF5 is higher than in normal conditions (Figure 8). This result indicates that the function of uORF3, uORF4, and uORF5 is regulated by uORF1 and uORF2 in order to allow the physiological levels of repression at the main coding sequence (Figure 8). The same result is observed in the absence of uORF1, uORF2, and uORF4 (Figure 8), indicating that uORF4 does not have a significant function in the translational regulation of the main coding sequence. However, we observe that in the absence of uORF1, uORF2, and uORF3, or in the absence of uORF1, uORF2, and uORF5, physiological levels of repression are obtained, indicating that among the five AUG uORFs, some are redundant. Together, these results support a model in which uORF1 (and to a less extent uORF2) functions as a ribosomal barrier to the efficient recognition of uORF3 and uORF5 initiation codons and thus inhibiting the full repressive function of these uORFs. After translation of uORF1 and uORF2, ribosomes may sometimes bypass uORF3 and uORF5 and will resume scanning to reinitiate translation at the main AUG, and thus contributing to the maintenance of the ABCE1 protein at physiological levels.

uORF-mediated translational regulation is well-characterized in transcripts that encode proteins involved in stress response [1,74]. This is the case of *ATF4* and *GCN4* transcripts, where the enhanced levels of eIF2α-P inhibit the formation of new and active ternary complexes in time to translate the inhibitory uORFs after translation of uORF1, leading to their bypass, and thus the 40S ribosomal subunit that remains attached to the mRNA, will resume scanning and reinitiate translation at the main ORF [72,73,75,76]. Although ABCE1 is not a stress-responsive protein, its uORFs seem to allow a slight translational derepression of the main ORF in conditions of ER stress induced by thapsigargin (Figure 9). uORFs are transversal to three different classes of transcripts facing high levels of eIF2α-P during thapsigargin-induced stress: (i) transcripts that are translationally induced, called “preferentially translated”; (ii) transcripts that are repressed; and (iii) transcripts that are resistant to eIF2α-P levels [37]. In general, it has been shown that the presence, number, and length of uORFs are not significantly distinct between the three groups of mRNAs; however, the uORF’s initiation codon context plays an important role in the translational control in ER stress [37]. It was demonstrated a prevalence of a weak Kozak context surrounding the uORF’s initiation codon in the preferential and resistant group of transcripts, together with a strong Kozak context for the main ORF, conversely to what occurs for the repression group of transcripts [37]. The fact that human *ABCE1* expression is tightly regulated by uORFs but slightly increases in stress conditions, and the fact that its AUG uORFs present initiation codons in suboptimal Kozak contexts, along with a strong Kozak context at the main AUG, places the *ABCE1* transcript as part of the preferentially translated group. We speculate that in stress conditions, after translation of ABCE1 uORF1, ribosomes might more efficiently bypass uORF3, uORF4, and uORF5 to reinitiate translation at the main AUG.

Although there is a correlation between uORF-mediated translational (dys)regulation and tumorigenesis [14,31], our data show that *ABCE1* uORF-mediated translational regulation does not seem to contribute to the tumorigenic process in HCT116 cells (Figure 10). Supporting the lack of oncogenic function of the *ABCE1* AUG uORFs, both ABCE1 mRNA and protein levels are similar in different colorectal cancer cell lines, as well as in the non-tumorigenic NCM460 cells (Figure 10). These results may indicate that, in colorectal cancer, ABCE1 does not behave as an oncogenic factor. Accordingly, Shichijo and co-workers provided evidence that *ABCE1* mRNA is ubiquitously expressed in normal and in cancer colorectal cells, including HCT116 [59]. Conversely, Hlavata and co-workers reported an upregulation of *ABCE1* mRNA in a pool of colorectal cancers [60]. However, the mRNA expression is not a direct indication of the protein expression since we need to consider the regulation at the translational level. In the Human Protein Atlas database, ABCE1 protein is expressed at similar levels in colorectal cancer tissues as well as in normal tissues, which agrees with our data.

In conclusion, our study identified so far unknown uORFs that tightly regulate translation of the human *ABCE1* transcript. This tight regulation contributes to maintaining approximately constant levels of ABCE1 protein in different cell environments.

## Figures and Tables

**Table 1:** Characterization of the AUG and non-AUG uORFs identified in the human *ABCE1* 5′-leader sequence.

uORF ID	uORF Nucleotide Sequence	uORF Length (nts)	Intercistronic Distance (nts)	uORF-Peptide Length (aa)	Kozak Sequence Context
uORF1	ATGACTAAGGCTCCACTCCTGACCCACCGGCCTTGA	36	426	11	GTTAGAATGA
uORF2	ATGCATCTGTTTACGCTAGGACCACGCTCGACGTCGGAGAAAAGCCCACACACTCACAGTTTCCAGACCTGGCTGCTTGCCGAAACTCAGATTCTCGGCACCTCCAGCAGCTGGCTTCGCCAACGGCGTTGA	132	252	43	AGGCGAATGC
uORF3	ATGACGTCATATCTCCCTACCTACCTCCAGGGTTCCGCCTCACGCTCTATGTCGCGCGCGCGCACTACGTCCTATGGCTTGCGCGTGCGGCGGCTGGGCACCGCCATTTTGGCCGGTGGCCGTGAGAACACGCTGTGTGGCTGA	144	91	47	AGCTCAATGA
uORF4	ATGTCGCGCGCGCGCACTACGTCCTATGGCTTGCGCGTGCGGCGGCTGGGCACCGCCATTTTGGCCGGTGGCCGTGAGAACACGCTGTGTGGCTGA	96	91	31	CGCTCTATGT
uORF5	ATGGCTTGCGCGTGCGGCGGCTGGGCACCGCCATTTTGGCCGGTGGCCGTGAGAACACGCTGTGTGGCTGAAAAGTGA	78	84	25	CGTCCTATGG
uORF6	GTGCGGCGGCTGGGCACCGCCATTTTGGCCGGTGGCCGTGAGAACACGCTGTGTGGCTGA	60	91	29	TTGCGCGTGC
uORF7	GTGGCCGTGAGAACACGCTGTGTGGCTGAAAAGTGA	36	84	11	TGGCCGGTGG
uORF8	GTGAAGGCAAGAGCTGATTTGGCCTCTGTGCTCCCCTCCGCAAGGGGATCGTTTTCTCCAGAAGAGCTGGATATTCTTTCGCCCAGTTATGGCAGACAAGTTAACGAGAATTGCTATTGTCAACCATGA	129	(overlapped)	42	TGAAAAGTGA
uORF9	GTGCTCCCCTCCGCAAGGGGATCGTTTTCTCCAGAAGAGCTGGATATTCTTTCGCCCAGTTATGGCAGACAAGTTAACGAGAATTGCTATTGTCAACCATGA	102	(overlapped)	33	GCCTCTGTGC
uORF10	CTGGATATTCTTTCGCCCAGTTATGGCAGACAAGTTAACGAGAATTGCTATTGTCAACCATGA	63	(overlapped)	20	GAAGAGCTGG

## Supplementary Materials

The following are available online at https://www.mdpi.com/article/10.3390/biomedicines9080911/s1, Table S1: Sequence of the primers used to generate the constructs needed to study the function of the AUG uORFs of the human *ABCE1* transcript., Table S2: Sequence of the primers used to generate the constructs needed to study the function of the non-AUG uORFs of the human *ABCE1* transcript., and Table S3: Primers used in the RT-qPCR and in the semi-quantitative RT-PCR.
