# Supplementary material for: Translation of ABCE1 Is Tightly Regulated by Upstream Open Reading Frames in Human Colorectal Cells
Source: Biomedicines. 2021 Jul 29;9(8):911. doi: 10.3390/biomedicines9080911 (PMC8389594; doi:10.3390/biomedicines9080911)
Supplement: Supplementary file 1 [file biomedicines-09-00911-s001.zip › biomedicines-1322743-supplementary.pdf]

SUPPLEMENTARY MATERIALS

Translation of *ABCE1* is tightly regulated by upstream open reading frames in human colorectal cells

Table S1. Sequence of the primers used to generate the constructs needed to study the function of the AUG uORFs of the human *ABCE1* transcript.

Primer	Sequence (5′→3′)
#1	CCCAAGCTTAAGCCGTGTCGGCACCAGAC
#2	CTTTATGTTTTTGGCGTCTTCCATAACTGGGCGAAAGAATATCC
#3	GGATATTCTTTCGCCCAGTTATGGAAGACGCCAAAAACATAAAG
#4	GGACTCTGGTACAAAATCGT
#5	CTGACACCTCCAGCGTTAGATTGACTAAGGCTCCACTCCTG
#6	CAGGAGTGGAGCCTTAGTCAATCTAACGCTGGAGGTGTCAG
#7	GATTGAAACCGGAGAGGCGATTGCATCTGTTTACGCTAGGA
#8	TCCTAGCGTAAACAGATGCAATCGCCTCTCCGGTTTCAATC
#9	TGAACAAGGGTCGCAGCTCATTGACGTCATATCTCCCTACC
#10	GGTAGGGAGATATGACGTCAATGAGCTGCGACCCTTGTTCA
#11	AGGGTTCCGCCTCACGCTCTTTGTCGCGCGCGCGCACTACG
#12	CGTAGTGCGCGCGCGCGACAAAGAGCGTGAGGCGGAACCCT
#13	GCGCGCGCGCACTACGTCCTTTGGCTTGCGCGTGCGGCGGC
#14	GCCGCCGCACGCGCAAGCCAAAGGACGTAGTGCGCGCGCGC
#15	TGGATATTCTTTCGCCCAGTATGGAAGACGCCAAAAACAT
#16	ATGTTTTTGGCGTCTTCCATACTGGGCGAAAGAATATCCA
#17	CACTCCTGACCCACCGGCCTGGAGGAAAGCGCAAACGTAAA
#18	TTTACGTTTGCGCTTTCCTCCAGGCCGGTGGGTCAGGAGTG
#19	GCTGGCTTCGCCAACGGCGTGGAACAAGGGTCGCAGCTCA
#20	TGAGCTGCGACCCTTGTTCCACGCCGTTGGCGAAGCCAGC
#21	CACGCTGTGTGGCTGAAAAGGGAAGGCAAGAGCTGATTTGG
#22	CCAAATCAGCTCTTGCCTTCCCTTTTCAGCCACACAGCGTG
#23	GAACAAGGGTCGCAGCTCAAGGACGTCATATCTCCCTACCT
#24	AGGTAGGGAGATATGACGTCCTTGAGCTGCGACCCTTGTTC
#25	GTGAGAACACGCTGTGTGGCGGAAAAGTGAAGGCAAGAGCT
#26	AGCTCTTGCCTTCACTTTTCCGCCACACAGCGTGTTCTCAC
#27	TGACACCTCCAGCGTTACCATGGCTAAGGCTCCACTCCTG
#28	CAGGAGTGGAGCCTTAGCCATGGTAACGCTGGAGGTGTCA
#29	GATTGAAACCGGAGAGGACCATGGATCTGTTTACGCTAGG
#30	CCTAGCGTAAACAGATCCATGGTCCTCTCCGGTTTCAATC
#31	TGAACAAGGGTCGCAGCACCATGGCGTCATATCTCCCTAC
#32	GTAGGGAGATATGACGCCATGGTGCTGCGACCCTTGTTCA
#33	AGGGTTCCGCCTCACGCACCATGGCGCGCGCGCGCACTAC
#34	GTAGTGCGCGCGCGCGCCATGGTGCGTGAGGCGGAACCCT
#35	GCGCGCGCGCACTACGTACCATGGCTTGCGCGTGCGG
#36	CCGCACGCGCAAGCCATGGTACGTAGTGCGCGCGCGC
#37	AAGAGCTGATTTGGCCTCTGGCTCCCCTCCGCAAGGGGAT
#38	ATCCCCTTGCGGAGGGGAGCCAGAGGCCAAATCAGCTCTT

Table S2. Sequence of the primers used to generate the constructs needed to study the function of the non-AUG uORFs of the human *ABCE1* transcript.

Primer	Sequence (5′→3′)
#1	GCGCGCGCGCACTACGTCCTTTGGCTTGCGCGCGCGGCGGCT
#2	AGCCGCCGCGCGCGCAAGCCAAAGGACGTAGTGCGCGCGCGC
#3	TACGTCCTATGGCTTGCGCGCGCGGCGGCTGGGCACCGCCA
#4	TGGCGGTGCCCAGCCGCCGCGCGCGCAAGCCATAGGACGTA
#5	GGCACCGCCATTTTGGCCGGCGGCCGTGAGAACACGCTGTG
#6	CACAGCGTGTTCTCACGGCCGCCGGCCAAAATGGCGGTGCC
#7	ACACGCTGTGTGGCTGAAAAGCGAAGGCAAGAGCTGATTTG
#8	CAAATCAGCTCTTGCCTTCGCTTTTCAGCCACACAGCGTGT
#9	CTGAAAAGTGAAGGCAAGAGGCGATTTGGCCTCTGTGCTCCC
#10	GGGAGCACAGAGGCCAAATCGCCTCTTGCCTTCACTTTTCAG
#11	CTGAAAAGCGAAGGCAAGAGGCGATTTGGCCTCTGTGCTCCC
#12	GGGAGCACAGAGGCCAAATCGCCTCTTGCCTTCGCTTTTCAG
#13	AAGAGGCGATTTGGCCTCTGCGCTCCCCTCCGCAAGGGGAT
#14	ATCCCCTTGCGGAGGGGAGCGCAGAGGCCAAATCGCCTCTT
#15	GATCGTTTTCTCCAGAAGAGGCGGATATTCTTTCGCCCAGTT
#16	AACTGGGCGAAAGAATATCCGCCTCTTCTGGAGAAAACGATC
#17	ACACGCTGTGTGGCTGAAAAATGAAGGCAAGAGCTGATTTG
#18	CAAATCAGCTCTTGCCTTCATTTTTCAGCCACACAGCGTGT
#19	CAAGAGCTGATTTGGCCTCTATGCTCCCCTCCGCAAGGGGA
#20	TCCCCTTGCGGAGGGGAGCATAGAGGCCAAATCAGCTCTTG
#21	GATCGTTTTCTCCAGAAGAGATGGATATTCTTTCGCCCAGT
#22	ACTGGGCGAAAGAATATCCATCTCTTCTGGAGAAAACGATC
#23	CTACGTCCTTTGGCTTGCGCATGCGGCGGCTGGGCACCGCC
#24	GGCGGTGCCCAGCCGCCGCATGCGCAAGCCAAAGGACGTAG
#25	GGGCACCGCCATTTTGGCCGATGGCCGTGAGAACACGCTGT
#26	ACAGCGTGTTCTCACGGCCATCGGCCAAAATGGCGGTGCCC

Table S3. Primers used in the RT-qPCR and in the semi-quantitative RT-PCR.

Primer	Sequence (5′→3′)
#1	CAACTGCATAAGGCTATGAAGAGA
#2	ATTTGTATTCAGCCCATATCGTTT
#3	AACGCGGCCTCTTCTTATTT
#4	ACCAGATTTGCCTGATTTGC
#5	ATTAAGAAATGCCCCTTTGGCG
#6	GAAGTTTGAAGGCATTGGCACA
#7	CCATGAGAAGTATGACAACAGCC
#8	GGGTGCTAAGCAGTTGGTG
